# Clonal Hematopoiesis: Updates and Implications at the Solid Tumor-Immune Interface

**DOI:** 10.1200/PO.23.00132

**Published:** 2023-06-21

**Authors:** Marco M. Buttigieg, Michael J. Rauh

**Affiliations:** ^1^ Department of Pathology and Molecular Medicine, Queen's University, Kingston, ON, Canada

## Abstract

Recent larger-scale studies of patients with cancer and longitudinal population cohorts have revealed how age-related expansions of mutant hematopoietic cells (clonal hematopoiesis [CH]) have differential associations with incident and prevalent cancers and their outcomes. Increasing recognition and deeper understanding of genetic subtypes of CH are yielding insights into the tumor-immune interface that may help to explain the heterogeneous impact of CH on tumorigenesis and treatment. Herein, we update the expanding influence of CH in precision oncology and propose important research and clinical questions to address to effectively manage and harness CH in oncology patients.

## Teaser

This article explores the potential consequences of clonal hematopoiesis for patients with solid tumor.

## INTRODUCTION

**FIG 1.**
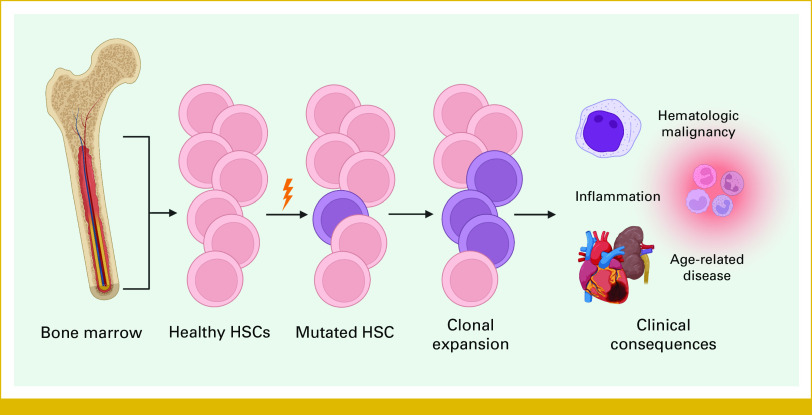
Schematic overview of clonal expansion of HSCs in CH and the associated clinical consequences of circulating CH-mutant HSC clones. After somatic mutations in a driver gene, the selective advantage created allows the HSC clone to expand in the bone marrow and become overrepresented in the blood, contributing to inflammation and age-related disease while posing a risk of malignant transformation. Created with BioRender.^4^ CH, clonal hematopoiesis; HSCs, hematopoietic stem cells.

Clonal hematopoiesis (CH) is the clonal expansion of hematopoietic stem cells (HSCs) and their progeny as driven by somatic mutations acquired during aging. CH describes any clonal expansions found in the blood and HSCs driven by somatic mutations and is thus inclusive of hematologic malignancies such as acute myeloid leukemia (AML) but typically refers to patients with premalignancy and no established disease.^1-3^ A second term, CH of indeterminate potential (CHIP), has been adopted to help differentiate between malignant and nonmalignant CH by specifically referring to the latter and its propensity, but indeterminate potential, for malignancy, and the absence of cytopenia (Fig 1). Between studies, the exact characterization of CH-driving mutations varies, but they are generally identified as cancer-driving single-nucleotide variants or smaller insertions or deletions (indels).^1-3^ The genes implicated with CH are largely drivers of myeloid hematologic malignancies. Most CH-causing mutations are found on three epigenetic regulator genes—*DNMT3A*, *TET2*, and *ASXL1*. Other frequently mutated gene targets include DNA damage response (DDR) genes such as *TP53* and *PPM1D*, cell growth signalers such as *JAK2* and *CBL*, and RNA splicing factors such as *SRSF2*, *SF3B1*, and *U2AF1*.^1-3,5,6^ More recent studies have also implicated lymphoid cancer–associated genes and mosaic chromosomal alterations (mCAs)—larger-scale genomic amplification, deletion, and loss of heterozygosity events—in the clonal expansion of HSCs; however, their integration into the literature is ongoing.^5,7-9^ Another defining characteristic of CH is variant allele frequency (VAF), which describes the fraction or percentage of DNA molecules sequenced that display a given mutation. There are no specific bounds to define CH, while the currently accepted VAF threshold for CHIP is at least 2%, indicating that 4% of total peripheral blood cells are affected, assuming a heterozygous mutation. Most studies have adopted this 2% VAF threshold for identifying CHIP and its corresponding clinical associations; however, this threshold is somewhat arbitrary and was initially determined by the standard limit of detection of next-generation sequencing (NGS) technologies. Newer technologies such as error-corrected (EC)-NGS can detect small CH clones with VAFs as low as 0.01% that normally escape detection, although the clinical implications of these clones should decrease with diminishing clone size.^1-3,10,11^ Owing to the lack of sufficient differentiation between CH and CHIP in most studies, we refer to both CH and CHIP as CH in this review for its broader definition.

**CONTEXT**

**Key Objective**
Clonal hematopoiesis (CH), the common age-associated expansion of somatically mutated blood cells, is associated with immune dysregulation, increased inflammatory disease, and hematologic malignancy risk. This review explores the implications of CH for solid cancers to provide recommendations for future research and practice.
**Knowledge Generated**
CH is common in patients with solid tumors, attributable to age, cancer treatments, and the possibility that CH is a risk factor for some cancers. Current applications in oncology include the optimization of tumor molecular profiling and management of therapy-related neoplasms. The effects of CH in the tumor microenvironment are diverse, varying between CH driver and cancer type.
**Relevance**
The frequent copresentation of CH with solid cancer presents a tremendous opportunity to improve clinical outcomes for patients with cancer. Implications of CH for some clinical applications are already clear, although much more research is needed to leverage CH as a tool in precision oncology.

Recent studies have revealed differential associations of CH with incident and prevalent cancers and their outcomes. Increasing recognition and deeper understanding of genetic subtypes of CH are yielding insights at the tumor-immune interface that may help to explain the heterogeneous impact of CH on tumorigenesis and treatment. Herein, we update the expanding influence of CH in precision oncology and propose important research and clinical questions to address in order to effectively manage and harness CH in oncology patients.

## CH IN INFLAMMATION AND AGE-RELATED DISEASE

CH is notable as a model of somatic mutations and aging in a variety of tissues, but its high prevalence and relationship with various inflammatory processes and age-related diseases makes it of great interest to clinicians and researchers. Initial population screenings for CH surveyed blood-derived genomic sequencing data from more than 30,000 individuals for evidence of clonal expansion. Together, these studies found that CH prevalence increases dramatically with age, detected in 10%-20% of individuals older than 70 years while being almost undetectable in those younger than 40 years. Of great concern, carriers of CH were found to have a 30%-40% increase in all-cause mortality.^1-3,6^ As noted previously, these estimates rely on older sequencing methods and higher VAF clones. With EC-NGS and the ability to detect lower VAF clones, <2% VAF CH appears almost ubiquitously in younger populations age 50-70 years, reaching rates of 95%, compared with 5% in the aforementioned studies.^11^

Although CH is a precursor state to hematologic malignancy, not all CH-mutant clones develop into cancer, and the risk of malignant transformation varies between subtypes of CH (reviewed in Bowman et al^12^). Initial studies revealed an approximately 10-fold increase in relative risk and 1% annual risk of malignant transformation, but it is again important to note the bias toward larger VAF clones because of study methodologies, and increasing CH clone size and the number of mutations were associated with increased risk of hematologic malignancy.^1-3^ Recent estimates using more sensitive sequencing methods predict a 3 to 5-fold increase in AML risk and when distinguishing between myeloid- and lymphoid-associated CH drivers, a 7-fold and 4.2-fold increase in relative risk of myeloid and lymphoid cancers, respectively.^5,13,14^ In addition to clone size, the risk of developing a hematologic malignancy is also modified by the affected gene(s). The two most commonly affected genes in CH, *DNMT3A* and *TET2* exhibit some of the lowest rates of AML progression while the less frequently mutated but still prevalent *TP53* and *U2AF1* demonstrate a much higher risk of malignant transformation and are associated with poorer AML prognosis.^1,13-15^ Certain mutations within genes also confer varying risks of progression, such as the *DNMT3A* R882H mutation—the most prevalent CH mutation overall—which is underrepresented in CH versus AML and myelodysplastic syndrome (MDS), indicating a potential for increased progression risk.^16^

Moving beyond the risk of overt hematologic malignancy, the altered inflammatory milieu imposed by CH promotes systemic inflammation and increased morbidity and mortality.^1,17-20^ Several studies of CH have characterized a hyperinflammatory environment, including elevated levels of tumor necrosis factor (TNF)-α, interleukin (IL)-6, and IL-1β through the activation of various inflammatory pathways.^1,17-20^ The effects of *DNMT3A* and *TET2* mutations have been best characterized in relation to inflammation, although knowledge is still incomplete. These epigenetic regulators have broad roles in restricting inflammation in the immune system, such as in monocyte/macrophages, where loss of function mutations in these genes promote the excessive release of proinflammatory cytokines.^1,17,21-23^ Importantly, the hyperinflammatory environment created by CH also acts alongside inflammatory stressors such as infection to promote the further development and expansion of CH clones, acting cyclically to exacerbate inflammation (reviewed in Cook et al^17^). For example, the elevated levels of TNF-α commonly seen in CH have been found to provide a fitness advantage for *TET2*-mutant HSCs, allowing them to continue to expand and proliferate to occupy a larger proportion of the active HSCs.^17,22,24,25^

A primary consequence of CH-associated inflammation and one of the main drivers of increased morbidity and mortality in CH carriers is an elevated risk of developing cardiovascular disease (CVD). Initially, the associations between CH and CVD were limited to coronary heart disease and ischemic stroke, where relative risk of incident events were, respectively, 2 and 2.5 in CH carriers, with mutations in *JAK2* specifically conferring a 12-fold risk increase for coronary heart disease.^1,2,26^ Subsequent cohort analyses and mechanistic studies have verified these initial findings while also expanding to show that CH is associated with increased risk of early-onset myocardial infarction, chronic heart failure, and thrombosis.^1,23,26-32^ CVD is not the only major clinical association of CH aside from hematologic malignancy. CH-associated inflammation has recently been linked to chronic kidney disease, with patients found to have elevated levels of CH versus the general population and had a more than doubled risk of kidney failure during the study follow-up period.^33^ Other adverse clinical outcomes associated with CH include autoimmune diseases such as antibody-associated vasculitis, chronic obstructive pulmonary disease, osteoporosis, and, albeit controversially, severe COVID-19.^34-38^ Curiously, the hyperinflammatory milieu conferred by CH may provide a protective effect in some diseases, such as in Alzheimer disease, where CH was found to reduce the risk for dementia and neuropathological features.^39^

## CURRENT INSIGHTS INTO CH AND SOLID CANCER

### Presence of CH in Patients With Solid Cancer

Several population cohort studies have already begun exploring another significant clinical association of CH—solid cancers. The first project specifically focused on CH in solid cancers was an analysis of 8,810 patients treated at Memorial Sloan Kettering (MSK). CH was common in this cohort, appearing in just over 25% of analyzed patients with solid cancer and associating with age, smoking, and previous exposure to therapy.^40^ Subsequent analyses of an overlapping but expanded cohort from MSK similarly found the prevalence of CH in patients with cancer to be 30%. Interestingly, the incident risk of CH was not uniform across cancer types—patients with thyroid and ovarian cancer demonstrated an elevated risk of CH, whereas melanoma, prostate cancer, colorectal cancer, and renal cell carcinomas were associated with a lower risk of CH.^41^ An additional analysis of cancer patient samples identified an increased risk of CH in thymoma patients and a reduced risk in bladder and breast cancers.^42^ The risk of confounding because of relationships with cancer treatment (see CH as a Predictor of Clinical Outcomes in Patients With Cancer section, below) and shared CH and cancer risk factors such as age and smoking limit the ability of these studies to make causal conclusions about the risk associations between CH and cancer.^41^

Larger, non–cancer-specific longitudinal studies have the added benefit of monitoring healthy people with CH over an extended period, better modeling incident disease risk. An analysis of 200,453 individuals enrolled in the UK Biobank study found various associations between CH status and the risk of developing a solid tumor. CH, especially with mutation VAF >10%, was linked with incident lung cancer, kidney cancer, lymphoma, and sarcoma. Notably, certain driver genes also carried their own incident risk associations—*DNMT3A*-mutant CH was additionally associated with incident stomach and bladder cancer while mutations in splicing factors *SF3B1* and *SRSF2* were uniquely linked with higher rates of colorectal and head/neck cancers.^43^ A similar analysis using 628,388 individuals from the UK Biobank found relationships between CH status and incident risk of lymphoid cancer, lung cancer in both smokers and nonsmokers, breast cancer, and prostate cancer.^44^ Strengthening the link between CH and lung cancer, CH conferred a 36% risk increase for disease across several cohorts, even when controlling for other confounders.^45^ Conversely, a recent evaluation of CH and prostate cancer risk found no association with the risk of prostate cancer.^46^

### CH as a Predictor of Clinical Outcomes in Patients With Cancer

**FIG 2.**
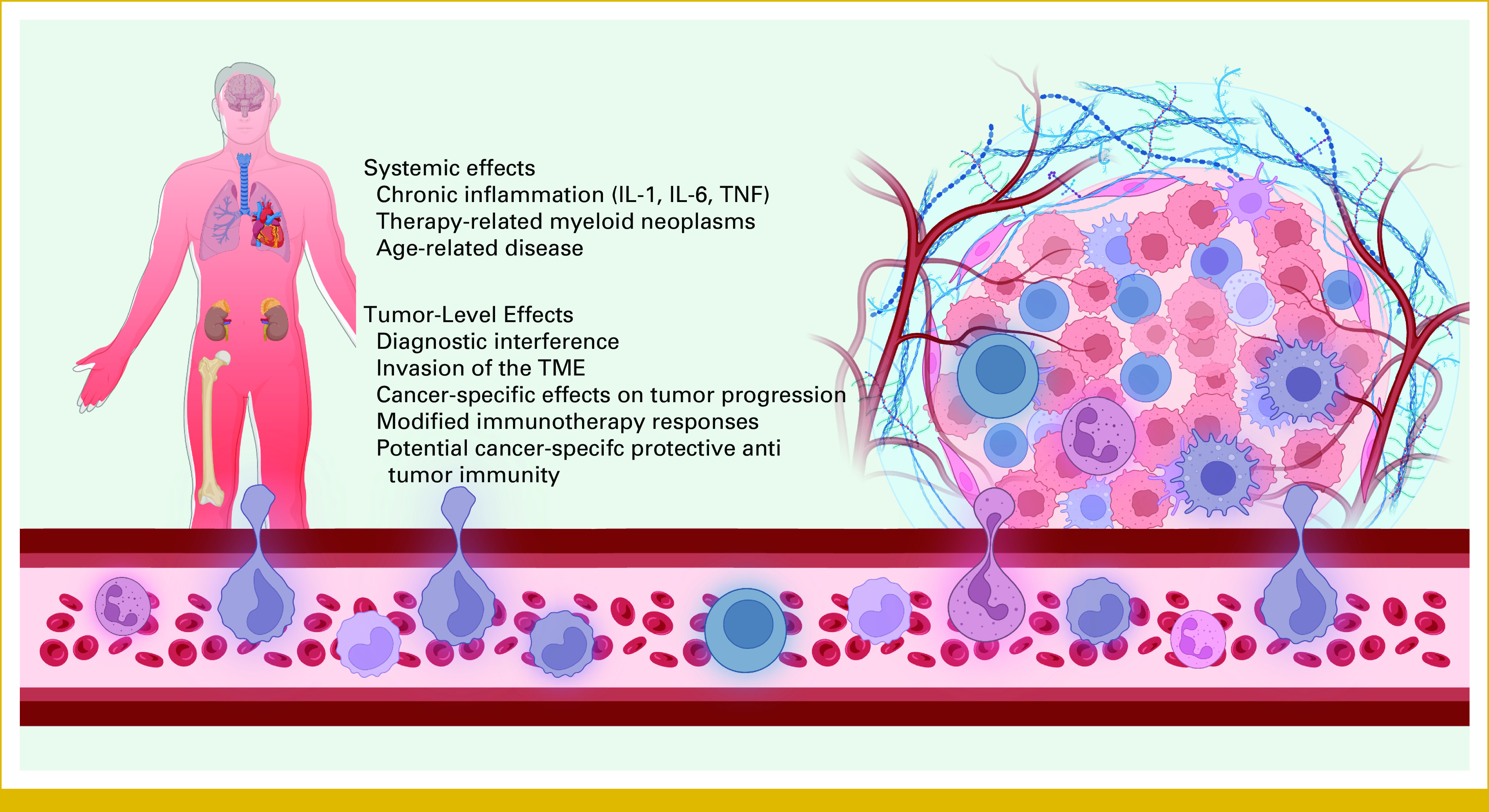
Summary of the systemic and tumor-level effects of CH in patients with solid tumor supported by recent research. Similar to healthy individuals, patients with cancer with CH will experience elevated systemic inflammation, higher risk of age-related conditions, especially cardiovascular disease, and increased hematologic cancer risk, primarily via therapy-related myeloid neoplasm. At the tumor level, CH has a diverse range of effects that vary on the basis of cancer type and CH driver mutation, while also interfering with the diagnosis of tumour mutations. Created with BioRender.^4^ CH, clonal hematopoiesis; IL, interleukin; TME, tumor microenvironment; TNF, tumor necrosis factor.

CH can play an important role in predicting various clinical and treatment outcomes after a cancer diagnosis (Fig 2). One of the better-studied implications of CH in this regard is the development of therapy-related myeloid neoplasms (tMNs)—a rare yet severe complication of cancer treatment. tMNs, which include AML, MDS, and MDS/myeloproliferative neoplasms, are gravely dangerous malignancies, with a 5-year survival of just 10%.^47^ It was previously thought that cancer therapies such as cytotoxic chemotherapy and radiotherapy were directly causing tMNs via mutagenic effects on HSCs; however, recent evidence has demonstrated that the presence of CH in patients with cancer significantly increases the likelihood of tMN development.^40,41,47^ In fact, the tMN driver mutations in adult patients with cancer are repeatedly found circulating before the receipt of cancer therapy, indicating that cancer therapy is instead providing a selective pressure that favors the growth and ultimately malignant transformation of preexisting CH clones.^41^ The effects of cancer therapy on CH clones are not uniform, differing on the basis of the role of the mutated driver gene. Studies investigating this phenomenon have recurrently found enrichment of DDR gene-mutant CH in patients after cytotoxic chemotherapy and radiotherapy, with experimental and clinical evidence supporting a fitness advantage for these mutations in the context of therapy.^40,41,48-51^ This may explain the previously noted enrichment of CH in some groups of patients with solid cancer, with treatments such as radioactive iodine and peptide receptor radionuclides implicated with increased prevalence of CH in thyroid and neuroendocrine tumors, respectively, although the potential roles of CH as a causal driver in tumorigenesis should not be neglected, as seen with lung cancers.^45,52,53^ Beyond the elevated risk of tMN development, cancer therapy–related CH driven by DDR gene mutations has also been implicated in other treatment complications via excessive inflammation, namely chemotherapy-induced cardiomyopathies and nonischemic heart failure.^54-56^ Although studies have found that immunotherapies generally have no influence on CH clones in patients with cancer, a case report highlighted a patient with large B-cell lymphoma who experienced the fatal expansion of a *TP53*-mutant CH clone after anti-CD19 chimeric antigen receptor (CAR) T-cell therapy, demonstrating a need for treatment-specific insights for immunotherapy and clonal dynamics in CH.^41,57^ In pediatric cancers, chemotherapy and radiation were found to predict CH presence in survivors, albeit these therapy-related clones remained stable in size during longitudinal follow-up.^58^

Patients with cancer with >10% VAF CH have been found to experience poorer overall survival than CH-negative patients, even when controlling for factors such as age, sex, and smoking. Strikingly, however, most of this effect was not driven by tMN development, but rather the most common cause of death among patients in the cohort was progression of the primary tumor.^40^ The reason for this effect on cancer progression is yet to be determined, with different cancer types and CH-driving mutations each experiencing distinct relationships (see CH at the Tumor-Immune Interface section, below). With tumor progression being a potential driver of this relationship between CH and mortality in patients with cancer, the lack of literature examining CH in cancer progression and metastasis is surprising. A small study of patients with metastatic renal cell carcinoma found CH in 43% of patients, negatively affecting overall survival.^59^ Sequencing of metastatic breast cancer specimens found that CH may be dictating bone metastasis, with enrichment for *DNMT3A* mutations in the samples found in a pattern resembling CH.^60^ Although these findings are notable, they are nonetheless incidental, and the conclusions that can be drawn from them are limited. A recent analysis of the results of the FIRE-3 trial for metastatic colorectal cancer was able to focus primarily on CH and outcomes in metastatic cancer, finding that 36% of the patients in the trial had CH and that CH was associated with *improved* survival outcomes—driven specifically by mutations in *DNMT3A*.^61^ In an effort to expand on these findings, the influence of CH on survival was evaluated in metastatic esophagogastric and colorectal cancers. This study found that CH was associated with reduced overall survival in the esophagogastric cancers and had a null effect in colorectal cancers—although *DNMT3A* mutation status was not independently evaluated as in the FIRE-3 trial analysis.^62^

CH may also provide value as a predictive biomarker for cancer immunotherapy treatments. A study conducted on the same MSK cohort described above examined the relationship between CH and outcomes for patients undergoing immune checkpoint inhibitor (ICI; anti–PD-1 receptor/ligand) therapy, finding that CH was predictive of poorer survival for most cancer types, with the notable exception of colorectal cancer.^63^ *DNMT3A* mutation status also identified a distinct subgroup of patients with metastatic non–small-cell lung cancer that saw improved responses to ICI, although it was unclear whether these mutations were found in tumor cells or CH-mutant tumor-infiltrating leukocytes.^64^ CH in CAR T-cell therapy has also demonstrated predictive value, although results vary between studies. An initial investigation showed that CH was associated with complete response and cytokine release syndrome but not survival in patients with non-Hodgkin lymphoma and multiple myeloma.^65^ Another study examining CAR T-cell therapy in non-Hodgkin lymphoma found contradicting results, with CH predicting improved overall survival but no change in response rates.^66^

### Diagnostic Relevance of CH in Solid Cancers

The complete range of prognostic and predictive implications of CH are yet to be fully realized; however, CH has already made an unplanned entry into precision oncology by appearing as an incidental finding in tumor or liquid biopsy genetic testing for patients. As discussed previously, the common mutational drivers of CH fall on common cancer driver genes, and as such, mutations carried by CH clones may be misinterpreted as tumor mutations or even germline events.

Germline interference is generally rare among patients with cancer—CH has been recorded to interfere in just 0.3% and 0.05% of patients in two large studies of germline genetic testing.^67,68^ Although rare, appropriate measures must be taken to avoid the misdiagnosis of germline conditions such as Li-Fraumeni syndrome (LFS), a condition driven by mutations in the common CH gene *TP53*. A follow-up study for *TP53* variants in germline testing was able to successfully distinguish LFS from CH and other somatic expansions using established LFS diagnostic criteria, tracking of variants through family history, and VAF evaluation.^69^

CH becomes slightly harder to differentiate when it is discovered alongside other somatic variants sourced from a patient's tumor. If both tumor and matched blood have been sequenced, this distinction can be informed by variations in VAF of the clone between the two samples, with higher blood VAF indicating a clone of hematopoietic origin.^40^ With tumor-only sequencing, CH mutations may lead to incorrect reporting of tumor variants, potentially leading to recommendations for inappropriate targeted therapies of little to no clinical benefit. In multiple cohorts, CH-associated mutations found in the blood are frequently detected in unpaired tumor sequencing, contributing to the erroneous calling of tumor variants in as many as 5% of patients.^70,71^ The contamination of liquid biopsies with CH variants is also problematic, with evaluations of cell-free DNA samples showing a significant proportion of somatic mutations that display features consistent with CH.^72-75^ As an incidental finding, CH with high-risk mutations can act as a point of referral for further hematologic consultation, helping to reveal occult hematological malignancies.^76^ The possibility of CH contaminating or interfering with diagnostic tests warrants consideration from precision oncologists, although knowledge of CH and the utilization of matched blood and/or tissue normal sequencing alongside tumor sequencing can drastically reduce the risk of any adverse consequences for patients.

### CH at the Tumor-Immune Interface

The clinical associations found between CH and cancer are becoming increasingly apparent with each upcoming study, so it is imperative that these are accompanied by basic research to support causal associations and identify potential confounding relationships. By using samples from primary breast tumors, researchers identified that immune cells carrying somatic mutations in CH driver genes were infiltrating the tumor microenvironment (TME).^77^ Additionally, there is evidence that patients with solid tumors with CH, at least those with *TET2* or *DNMT3A* variants, experience elevated levels of lymphocyte invasion in the TME.^78^ Given that these mutant immune cells are entering the TME, it is reasonable to predict that they might be disturbing the intricate balance of immunity and eliciting a direct effect on the growth and progression of the tumor.

At the cellular level, changes to immune function induced by CH driver mutations have both protumorigenic and antitumorigenic effects. For example, macrophage-specific mutations in *TET2* and *PPM1D* can drive elevated levels of IL-1β and IL-18 via increased activation of the NLRP3 inflammasome.^32,56^ The NLRP3 inflammasome has been shown to play a bidirectional role in the antitumor immune response, with activation of the pathway linked to the progression of several types of cancer such as breast cancer, lung cancer, and lymphoma, although of note, NLRP3 activation has been found to be protective in colorectal cancers.^79-83^ IL-6, another commonly elevated inflammatory marker in CH, is linked with not only numerous detrimental processes in tumorigenesis such as tumor cell proliferation and angiogenesis but also antitumorigenic processes such as T-cell trafficking to the tumor site.^84^ The disruption of common CH driver genes, namely *DNMT3A* and *TET2*, can also directly modulate the function of CAR T cells. With the loss of *TET2*, CAR T cells demonstrated a central memory phenotype that helped enhance the potency of the cells while *DNMT3A* regularly plays a role in inducing the epigenetic changes that underlie exhaustion, so deletion facilitated an enhanced antitumor response.^85,86^ These studies directly reference CH mutations in CAR T cells, although it is yet to be determined how these mutations in normal circulating human T cells can modulate their function.

Animal models have also provided evidence for distinct roles of the CH driver genes in tumorigenesis. *TET2* has been most extensively studied in this regard, with one study highlighting a protumorigenic effect of *TET2* deletion that operates through increased populations of granulocytic myeloid-derived suppressor cells that deplete CD8+ T cells, driving immunosuppression and tumor growth in models of hepatocellular carcinoma and breast cancer.^87^ Other models of hematopoietic *TET2* depletion have shown varying effects, promoting angiogenesis and tumor progression in lung cancer while reducing tumor burden in melanoma by facilitating a proinflammatory tumor-associated macrophage phenotype that augmented T-cell infiltration.^88,89^ Less data are available for other common CH drivers, but similar trends appear. *DNMT3A* loss of function mutations appeared to drive colitis-associated colon cancer growth and progression, although the exact mechanism has not yet been elucidated.^90^ *ASXL1* mutations were found to promote tumorigenesis in a variety of cancer models through disrupted T-cell development and functionality.^91^ Finally, wild-type p53 in myeloid cells was found to suppress M2 macrophage polarization and tumor invasiveness in an intestinal cancer model, indicating a potential cancer risk with *TP53* loss-of-function mutations in CH.^92^

## FUTURE DIRECTIONS AND CHALLENGES

The findings presented here depict an exciting future for research examining the role of CH in solid tumors. Building on this foundation, there are a number of additional steps that need to be taken to translate these findings into precision oncology practice. Primarily, current knowledge of CH in solid cancers depicts a relationship that is as heterogeneous as cancer itself, and more research will be required to better understand this relationship to determine the contexts where CH is helpful, harmful, both, or neutral for patients with solid tumors. Further analyses of longitudinal population and cancer-specific cohorts will provide a better estimate of the risk of specific cancers and clinical outcomes in relation to CH, hopefully while being able to separate confounding effects due to cancer therapy and shared risk factors between both conditions. Although connected by signs of systemic inflammation and immune dysfunction, the variety of CH driver mutations have demonstrated diverse effects on tumorigenesis at different sites, the most notable of which being the distinct protective effect of CH reported in colorectal cancers. These nuances must be studied further if we ever hope to use CH in cancer therapy—both to develop novel strategies and optimize existing ones, whether that be targeting CH directly, incorporating CH as a prognostic and/or predictive biomarker, or leveraging CH in the TME to our benefit.

Targeting CH directly remains elusive, largely driven by a lack of motivation for clinical trials because of the limited risk-benefit profile that has been proposed by existing research. Potential avenues of treatment have been reviewed previously with relevance to precision oncology by Miller and Steensma,^93^ although some novel approaches have been proposed since then. For example, *DNMT3A* R882 mutations are noted as the most prevalent of all CH variants across many studies, and the herbal extract, oridonin, has been proposed as a promising candidate to suppress both CH and leukemias that are driven by such mutations.^94^ One novel approach for *TET2*-mutant CH is the utilization of eltrombopag, a thrombopoietin receptor agonist that can restrict the growth of malignant *TET2-*mutant clones while favoring the expansion of healthy cells.^95^ Targeting of *TET2-*mutant cells has also shown promise with the mutant *IDH1/2* metabolite 2-hydroxyglutarate and similarly engineered small molecule inhibitors, as well as *XPO1* inhibitors.^96,97^ Other advancements include the potential of *PARP1* inhibition for antagonizing *TET2*-mutant CH; however, the risk of hematologic malignancy is elevated in patients undergoing this therapy and thus more research is warranted before this can be applied safely in a clinical setting.^44,98^ With the implication of inflammasome activity in *TET2*-mutant and other forms of CH, clinical trials such as IMPACT are now investigating the value of the anti–IL-1β antibody canakinumab in high-risk CH (ie, clonal cytopenia of undetermined significance; ClinicalTrials.gov identifier: NCT05641831).

Beyond cancer, developments in our understanding of the biology of CH will also prove beneficial in a variety of clinical settings. For example, the implications of more novel CH drivers such as lymphoid cancer–associated genes and mCAs are not yet fully elucidated in a more general sense, let alone in the context of cancer. Looking toward the future, the influence of CH in the era of precision medicine is expanding rapidly, and patients with solid cancer await the research and development of effective strategies for managing CH in a clinical oncology setting.

## References

[1] Jaiswal S Ebert BL Clonal hematopoiesis in human aging and disease Science 366 eaan4673 2019 31672865 10.1126/science.aan4673 PMC8050831

[2] Jaiswal S Fontanillas P Flannick J et al Age-related clonal hematopoiesis associated with adverse outcomes N Engl J Med 371 2488 2498 2014 25426837 10.1056/NEJMoa1408617 PMC4306669

[3] Genovese G Kähler AK Handsaker RE et al Clonal hematopoiesis and blood-cancer risk inferred from blood DNA sequence N Engl J Med 371 2477 2487 2014 25426838 10.1056/NEJMoa1409405 PMC4290021

[4] BioRender. http://biorender.com

[5] Niroula A Sekar A Murakami MA et al Distinction of lymphoid and myeloid clonal hematopoiesis Nat Med 27 1921 1927 2021 34663986 10.1038/s41591-021-01521-4 PMC8621497

[6] Xie M Lu C Wang J et al Age-related mutations associated with clonal hematopoietic expansion and malignancies Nat Med 20 1472 1478 2014 25326804 10.1038/nm.3733 PMC4313872

[7] Saiki R Momozawa Y Nannya Y et al Combined landscape of single-nucleotide variants and copy number alterations in clonal hematopoiesis Nat Med 27 1239 1249 2021 34239136 10.1038/s41591-021-01411-9

[8] Loh P-R Genovese G McCarroll SA Monogenic and polygenic inheritance become instruments for clonal selection Nature 584 136 141 2020 32581363 10.1038/s41586-020-2430-6 PMC7415571

[9] Gao T Ptashkin R Bolton KL et al Interplay between chromosomal alterations and gene mutations shapes the evolutionary trajectory of clonal hematopoiesis Nat Commun 12 338 2021 33436578 10.1038/s41467-020-20565-7 PMC7804935

[10] Steensma DP Bejar R Jaiswal S et al Clonal hematopoiesis of indeterminate potential and its distinction from myelodysplastic syndromes Blood 126 9 16 2015 25931582 10.1182/blood-2015-03-631747 PMC4624443

[11] Young AL Challen GA Birmann BM et al Clonal haematopoiesis harbouring AML-associated mutations is ubiquitous in healthy adults Nat Commun 7 12484 2016 27546487 10.1038/ncomms12484 PMC4996934

[12] Bowman RL Busque L Levine RL Clonal hematopoiesis and evolution to hematopoietic malignancies Cell Stem Cell 22 157 170 2018 29395053 10.1016/j.stem.2018.01.011 PMC5804896

[13] Abelson S Collord G Ng SWK et al Prediction of acute myeloid leukaemia risk in healthy individuals Nature 559 400 404 2018 29988082 10.1038/s41586-018-0317-6 PMC6485381

[14] Desai P Mencia-Trinchant N Savenkov O et al Somatic mutations precede acute myeloid leukemia years before diagnosis Nat Med 24 1015 1023 2018 29988143 10.1038/s41591-018-0081-z PMC6849383

[15] Papaemmanuil E Gerstung M Bullinger L et al Genomic classification and prognosis in acute myeloid leukemia N Engl J Med 374 2209 2221 2016 27276561 10.1056/NEJMoa1516192 PMC4979995

[16] Buscarlet M Provost S Zada YF et al DNMT3A and TET2 dominate clonal hematopoiesis and demonstrate benign phenotypes and different genetic predispositions Blood 130 753 762 2017 28655780 10.1182/blood-2017-04-777029

[17] Cook EK Luo M Rauh MJ Clonal hematopoiesis and inflammation: Partners in leukemogenesis and comorbidity Exp Hematol 83 85 94 2020 32001341 10.1016/j.exphem.2020.01.011

[18] Cook EK Izukawa T Young S et al Comorbid and inflammatory characteristics of genetic subtypes of clonal hematopoiesis Blood Adv 3 2482 2486 2019 31434682 10.1182/bloodadvances.2018024729 PMC6712533

[19] Trowbridge JJ Starczynowski DT Innate immune pathways and inflammation in hematopoietic aging, clonal hematopoiesis, and MDS J Exp Med 218 e20201544 2021 34129017 10.1084/jem.20201544 PMC8210621

[20] Yura Y Sano S Walsh K Clonal hematopoiesis: A new step linking inflammation to heart failure JACC Basic Transl Sci 5 196 207 2020 32140625 10.1016/j.jacbts.2019.08.006 PMC7046537

[21] Cobo I Tanaka T Glass CK et al Clonal hematopoiesis driven by DNMT3A and TET2 mutations: Role in monocyte and macrophage biology and atherosclerotic cardiovascular disease Curr Opin Hematol 29 1 7 2022 34654019 10.1097/MOH.0000000000000688 PMC8639635

[22] Cull AH Snetsinger B Buckstein R et al Tet2 restrains inflammatory gene expression in macrophages Exp Hematol 55 56 70.e13 2017 28826859 10.1016/j.exphem.2017.08.001

[23] Sano S Oshima K Wang Y et al CRISPR-mediated gene editing to assess the roles of Tet2 and Dnmt3a in clonal hematopoiesis and cardiovascular disease Circ Res 123 335 341 2018 29728415 10.1161/CIRCRESAHA.118.313225 PMC6054544

[24] Hormaechea-Agulla D Matatall KA Le DT et al Chronic infection drives Dnmt3a-loss-of-function clonal hematopoiesis via IFNγ signaling Cell Stem Cell 28 1428 1442.e6 2021 33743191 10.1016/j.stem.2021.03.002 PMC8349829

[25] Abegunde SO Buckstein R Wells RA et al An inflammatory environment containing TNFα favors Tet2-mutant clonal hematopoiesis Exp Hematol 59 60 65 2018 29195897 10.1016/j.exphem.2017.11.002

[26] Jaiswal S Natarajan P Silver AJ et al Clonal hematopoiesis and risk of atherosclerotic cardiovascular disease N Engl J Med 377 111 121 2017 28636844 10.1056/NEJMoa1701719 PMC6717509

[27] Dorsheimer L Assmus B Rasper T et al Association of mutations contributing to clonal hematopoiesis with prognosis in chronic ischemic heart failure JAMA Cardiol 4 25 2019 30566180 10.1001/jamacardio.2018.3965 PMC6439691

[28] Wolach O Sellar RS Martinod K et al Increased neutrophil extracellular trap formation promotes thrombosis in myeloproliferative neoplasms Sci Transl Med 10 eaan8292 2018 29643232 10.1126/scitranslmed.aan8292 PMC6442466

[29] Marnell CS Bick A Natarajan P Clonal hematopoiesis of indeterminate potential (CHIP): Linking somatic mutations, hematopoiesis, chronic inflammation and cardiovascular disease J Mol Cell Cardiol 161 98 105 2021 34298011 10.1016/j.yjmcc.2021.07.004 PMC8629838

[30] Abplanalp WT Mas-Peiro S Cremer S et al Association of clonal hematopoiesis of indeterminate potential with inflammatory gene expression in patients with severe degenerative aortic valve stenosis or chronic postischemic heart failure JAMA Cardiol 5 1170 1175 2020 32639511 10.1001/jamacardio.2020.2468 PMC7344831

[31] Fuster JJ MacLauchlan S Zuriaga MA et al Clonal hematopoiesis associated with TET2 deficiency accelerates atherosclerosis development in mice Science 355 842 847 2017 28104796 10.1126/science.aag1381 PMC5542057

[32] Sano S Oshima K Wang Y et al Tet2-mediated clonal hematopoiesis accelerates heart failure through a mechanism involving the IL-1β/NLRP3 inflammasome J Am Coll Cardiol 71 875 886 2018 29471939 10.1016/j.jacc.2017.12.037 PMC5828038

[33] Vlasschaert C McNaughton AJM Chong M et al Association of clonal hematopoiesis of indeterminate potential with worse kidney function and anemia in two cohorts of patients with advanced chronic kidney disease J Am Soc Nephrol 33 985 995 2022 35197325 10.1681/ASN.2021060774 PMC9063886

[34] Arends CM Weiss M Christen F et al Clonal hematopoiesis in patients with anti-neutrophil cytoplasmic antibody-associated vasculitis Haematologica 105 e264 e267 2020 31582546 10.3324/haematol.2019.223305 PMC7271609

[35] Miller PG Qiao D Rojas-Quintero J et al Association of clonal hematopoiesis with chronic obstructive pulmonary disease Blood 139 357 368 2022 34855941 10.1182/blood.2021013531 PMC8777202

[36] Kim PG Niroula A Shkolnik V et al Dnmt3a-mutated clonal hematopoiesis promotes osteoporosis J Exp Med 218 e20211872 2021 34698806 10.1084/jem.20211872 PMC8552148

[37] Bolton KL Koh Y Foote MB et al Clonal hematopoiesis is associated with risk of severe Covid-19 Nat Commun 12 5975 2021 34645798 10.1038/s41467-021-26138-6 PMC8514469

[38] Zhou Y Shalhoub RN Rogers SN et al Clonal hematopoiesis is not significantly associated with Covid-19 disease severity Blood 140 14 2022 10.1182/blood.2022015721 PMC9293387 35839449

[39] Bouzid H Belk JA Jan M et al Clonal hematopoiesis is associated with protection from Alzheimer’s disease. medRxiv 10.1101/2021.12.10.21267552v1 PMC10353941 37322115

[40] Coombs CC Zehir A Devlin SM et al Therapy-related clonal hematopoiesis in patients with non-hematologic cancers is common and associated with adverse clinical outcomes Cell Stem Cell 21 374 382.e4 2017 28803919 10.1016/j.stem.2017.07.010 PMC5591073

[41] Bolton KL Ptashkin RN Gao T et al Cancer therapy shapes the fitness landscape of clonal hematopoiesis Nat Genet 52 1219 1226 2020 33106634 10.1038/s41588-020-00710-0 PMC7891089

[42] Pich O Reyes-Salazar I Gonzalez-Perez A et al Discovering the drivers of clonal hematopoiesis Nat Commun 13 4267 2022 35871184 10.1038/s41467-022-31878-0 PMC9308779

[43] Kar SP Quiros PM Gu M et al Genome-wide analyses of 200,453 individuals yield new insights into the causes and consequences of clonal hematopoiesis Nat Genet 54 1155 1166 2022 35835912 10.1038/s41588-022-01121-z PMC9355874

[44] Kessler MD Damask A O’Keeffe S et al Common and rare variant associations with clonal haematopoiesis phenotypes Nature 612 301 309 2022 36450978 10.1038/s41586-022-05448-9 PMC9713173

[45] Tian R Wiley B Liu J et al Clonal hematopoiesis and risk of incident lung cancer J Clin Oncol 41 1423 1433 2023 36480766 10.1200/JCO.22.00857 PMC9995101

[46] Wang A Xu Y Yu Y et al Clonal hematopoiesis and risk of prostate cancer in large samples of European ancestry men Hum Mol Genet 32 489 495 2022 10.1093/hmg/ddac214 PMC9851740 36018819

[47] McNerney ME Godley LA Le Beau MM Therapy-related myeloid neoplasms: When genetics and environment collide Nat Rev Cancer 17 513 527 2017 28835720 10.1038/nrc.2017.60 PMC5946699

[48] Sperling AS Guerra VA Kennedy JA et al Lenalidomide promotes the development of TP53-mutated therapy-related myeloid neoplasms Blood 140 1753 1763 2022 35512188 10.1182/blood.2021014956 PMC9837415

[49] Hsu JI Dayaram T Tovy A et al PPM1D mutations drive clonal hematopoiesis in response to cytotoxic chemotherapy Cell Stem Cell 23 700 713.e6 2018 30388424 10.1016/j.stem.2018.10.004 PMC6224657

[50] Kahn JD Miller PG Silver AJ et al PPM1D-truncating mutations confer resistance to chemotherapy and sensitivity to PPM1D inhibition in hematopoietic cells Blood 132 1095 1105 2018 29954749 10.1182/blood-2018-05-850339 PMC6137556

[51] Wong TN Ramsingh G Young AL et al Role of TP53 mutations in the origin and evolution of therapy-related acute myeloid leukaemia Nature 518 552 555 2015 25487151 10.1038/nature13968 PMC4403236

[52] Boucai L Falcone J Ukena J et al Radioactive iodine–related clonal hematopoiesis in thyroid cancer is common and associated with decreased survival J Clin Endocrinol Metab 103 4216 4223 2018 30137527 10.1210/jc.2018-00803 PMC6194804

[53] Singh A Mencia-Trinchant N Griffiths EA et al Mutant PPM1D- and TP53-driven hematopoiesis populates the hematopoietic compartment in response to peptide receptor radionuclide therapy JCO Precis Oncol 10.1200/PO.21.00309 2022 PMC8769150 35025619

[54] Evans MA Sano S Walsh K Cardiovascular disease, aging, and clonal hematopoiesis Annu Rev Pathol Mech Dis 15 419 438 2020 10.1146/annurev-pathmechdis-012419-032544 PMC7104598 31689371

[55] Sano S Wang Y Ogawa H et al TP53-mediated therapy-related clonal hematopoiesis contributes to doxorubicin-induced cardiomyopathy by augmenting a neutrophil-mediated cytotoxic response JCI Insight 6 e146076 2021 34236050 10.1172/jci.insight.146076 PMC8410064

[56] Yura Y Miura-Yura E Katanasaka Y et al The cancer therapy-related clonal hematopoiesis driver gene Ppm1d promotes inflammation and non-ischemic heart failure in mice Circ Res 129 684 698 2021 34315245 10.1161/CIRCRESAHA.121.319314 PMC8409899

[57] Eder LN Martinovic D Mazzeo P et al Fatal progression of mutated TP53-associated clonal hematopoiesis following anti-CD19 CAR-T cell therapy Curr Oncol 30 1146 1150 2023 36661736 10.3390/curroncol30010087 PMC9858310

[58] Hagiwara K Natarajan S Wang Z et al Dynamics of age- versus therapy-related clonal hematopoiesis in long-term survivors of pediatric cancer Cancer Discov 13 844 857 2023 36751942 10.1158/2159-8290.CD-22-0956 PMC10070170

[59] Bacon JVW Annala M Soleimani M et al Plasma circulating tumor DNA and clonal hematopoiesis in metastatic renal cell carcinoma Clin Genitourin Cancer 18 322 331.e2 2020 32046920 10.1016/j.clgc.2019.12.018

[60] Rinaldi J Sokol ES Hartmaier RJ et al The genomic landscape of metastatic breast cancer: Insights from 11,000 tumors PLoS One 15 e0231999 2020 32374727 10.1371/journal.pone.0231999 PMC7202592

[61] Arends CM Dimitriou S Stahler A et al Clonal hematopoiesis is associated with improved survival in patients with metastatic colorectal cancer from the FIRE-3 trial Blood 139 1593 1597 2022 34932794 10.1182/blood.2021014108

[62] Diplas BH Ptashkin R Chou JF et al Clinical importance of clonal hematopoiesis in metastatic gastrointestinal tract cancers JAMA Netw Open 6 e2254221 2023 36729457 10.1001/jamanetworkopen.2022.54221 PMC9896303

[63] Hsiehchen D Sfreddo HJ Zhao K et al Clonal hematopoiesis and differential outcomes after immune checkpoint blockade Cancer Cell 40 1071 1072 2022 36113476 10.1016/j.ccell.2022.08.024 PMC11660592

[64] Ricciuti B Alessi JVM Li YY et al DNMT3A mutation to identify a subset of non-small cell lung cancers with increased sensitivity to PD-(L)1 blockade J Clin Oncol 39 2021 suppl 15; abstr 9113

[65] Miller PG Sperling AS Brea EJ et al Clonal hematopoiesis in patients receiving chimeric antigen receptor T-cell therapy Blood Adv 5 2982 2986 2021 34342642 10.1182/bloodadvances.2021004554 PMC8361461

[66] Teipel R Kroschinsky F Kramer M et al Prevalence and variation of CHIP in patients with aggressive lymphomas undergoing CD19-directed CAR T-cell treatment Blood Adv 6 1941 1946 2022 35008107 10.1182/bloodadvances.2021005747 PMC8941459

[67] Maani N Panabaker K McCuaig JM et al Incidental findings from cancer next generation sequencing panels npj Genomic Med 6 63 2021 10.1038/s41525-021-00224-6 PMC8289933 34282142

[68] Weitzel JN Chao EC Nehoray B et al Somatic TP53 variants frequently confound germ-line testing results Genet Med 20 809 816 2018 29189820 10.1038/gim.2017.196 PMC5976505

[69] Schwartz AN Hyman SR Stokes SM et al Evaluation of TP53 variants detected on peripheral blood or saliva testing: Discerning germline from somatic TP53 variants JCO Precis Oncol 10.1200/PO.21.00278 2021 34994652

[70] Ptashkin RN Mandelker DL Coombs CC et al Prevalence of clonal hematopoiesis mutations in tumor-only clinical genomic profiling of solid tumors JAMA Oncol 4 1589 1593 2018 29872864 10.1001/jamaoncol.2018.2297 PMC6224316

[71] Coombs CC Gillis NK Tan X et al Identification of clonal hematopoiesis mutations in solid tumor patients undergoing unpaired next-generation sequencing assays Clin Cancer Res 24 5918 5924 2018 29866652 10.1158/1078-0432.CCR-18-1201 PMC6812550

[72] Razavi P Li BT Brown DN et al High-intensity sequencing reveals the sources of plasma circulating cell-free DNA variants Nat Med 25 1928 1937 2019 31768066 10.1038/s41591-019-0652-7 PMC7061455

[73] Hu Y Ulrich BC Supplee J et al False-positive plasma genotyping due to clonal hematopoiesis Clin Cancer Res 24 4437 4443 2018 29567812 10.1158/1078-0432.CCR-18-0143

[74] Jensen K Konnick EQ Schweizer MT et al Association of clonal hematopoiesis in DNA repair genes with prostate cancer plasma cell-free DNA testing interference JAMA Oncol 7 107 2021 33151258 10.1001/jamaoncol.2020.5161 PMC7645740

[75] Kotecha RR Gedvilaite E Ptashkin R et al Matched molecular profiling of cell-free DNA and tumor tissue in patients with advanced clear cell renal cell carcinoma JCO Precis Oncol 10.1200/PO.22.00012 2022 PMC9489165 35797508

[76] Aldea M Tagliamento M Bayle A et al Liquid biopsies for circulating tumor DNA detection may reveal occult hematologic malignancies in patients with solid tumors JCO Precis Oncol 10.1200/PO.22.00583 2023 36862966

[77] Kleppe M Comen E Wen HY et al Somatic mutations in leukocytes infiltrating primary breast cancers npj Breast Cancer 1 15005 2015 28721364 10.1038/npjbcancer.2015.5 PMC5515194

[78] Severson EA Riedlinger GM Connelly CF et al Detection of clonal hematopoiesis of indeterminate potential in clinical sequencing of solid tumor specimens Blood 131 2501 2505 2018 29678827 10.1182/blood-2018-03-840629 PMC5981171

[79] Hamarsheh S Zeiser R NLRP3 inflammasome activation in cancer: A double-edged sword Front Immunol 11 1444 2020 32733479 10.3389/fimmu.2020.01444 PMC7360837

[80] Guo B Fu S Zhang J et al Targeting inflammasome/IL-1 pathways for cancer immunotherapy Sci Rep 6 36107 2016 27786298 10.1038/srep36107 PMC5082376

[81] Wang Y Kong H Zeng X et al Activation of NLRP3 inflammasome enhances the proliferation and migration of A549 lung cancer cells Oncol Rep 35 2053 2064 2016 26782741 10.3892/or.2016.4569

[82] Zhao X Zhang C Hua M et al NLRP3 inflammasome activation plays a carcinogenic role through effector cytokine IL-18 in lymphoma Oncotarget 8 108571 108583 2017 29312552 10.18632/oncotarget.21010 PMC5752465

[83] Dupaul-Chicoine J Arabzadeh A Dagenais M et al The Nlrp3 inflammasome suppresses colorectal cancer metastatic growth in the liver by promoting natural killer cell tumoricidal activity Immunity 43 751 763 2015 26384545 10.1016/j.immuni.2015.08.013

[84] Fisher DT Appenheimer MM Evans SS The two faces of IL-6 in the tumor microenvironment Semin Immunol 26 38 47 2014 24602448 10.1016/j.smim.2014.01.008 PMC3970580

[85] Fraietta JA Nobles CL Sammons MA et al Disruption of TET2 promotes the therapeutic efficacy of CD19-targeted T cells Nature 558 307 312 2018 29849141 10.1038/s41586-018-0178-z PMC6320248

[86] Prinzing B Zebley CC Petersen CT et al Deleting DNMT3A in CAR T cells prevents exhaustion and enhances antitumor activity Sci Transl Med 13 eabh0272 2021 34788079 10.1126/scitranslmed.abh0272 PMC8733956

[87] Li S Feng J Wu F et al TET2 promotes anti-tumor immunity by governing G-MDSCs and CD8+ T-cell numbers EMBO Rep 21 e49425 2020 32929842 10.15252/embr.201949425 PMC7534639

[88] Nguyen YTM Fujisawa M Nguyen TB et al Tet2 deficiency in immune cells exacerbates tumor progression by increasing angiogenesis in a lung cancer model Cancer Sci 112 4931 4943 2021 34657351 10.1111/cas.15165 PMC8645781

[89] Pan W Zhu S Qu K et al The DNA methylcytosine dioxygenase Tet2 sustains immunosuppressive function of tumor-infiltrating myeloid cells to promote melanoma progression Immunity 47 284 297.e5 2017 28813659 10.1016/j.immuni.2017.07.020 PMC5710009

[90] Feng Y Newsome R Robinson T et al Dnmt3a mutations in the hematopoietic system promote colitis-associated colon cancer: A model of clonal hematopoiesis in solid tumors Blood 138 2021 suppl 1; abstr 2161

[91] Liu X Sato N Shimosato Y et al CHIP-associated mutant ASXL1 in blood cells promotes solid tumor progression Cancer Sci 113 1182 1194 2022 35133065 10.1111/cas.15294 PMC8990791

[92] He X-Y Xiang C Zhang C-X et al p53 in the myeloid lineage modulates an inflammatory microenvironment limiting initiation and invasion of intestinal tumors Cell Rep 13 888 897 2015 26565902 10.1016/j.celrep.2015.09.045

[93] Miller PG Steensma DP Implications of clonal hematopoiesis for precision oncology JCO Precis Oncol 10.1200/PO.20.00144 2020 35050749

[94] Liao M Dong Q Chen R et al Oridonin inhibits DNMT3A R882 mutation-driven clonal hematopoiesis and leukemia by inducing apoptosis and necroptosis Cell Death Discov 7 297 2021 34663800 10.1038/s41420-021-00697-5 PMC8523644

[95] Guan Y Hasipek M Jiang D et al Eltrombopag inhibits TET dioxygenase to contribute to hematopoietic stem cell expansion in aplastic anemia J Clin Invest 132 e149856 2022 35085104 10.1172/JCI149856 PMC8843742

[96] Guan Y Tiwari AD Phillips JG et al A therapeutic strategy for preferential targeting of TET2-mutant and TET dioxygenase–deficient cells in myeloid neoplasms Blood Cancer Discov 2 146 161 2021 33681816 10.1158/2643-3230.BCD-20-0173 PMC7935131

[97] Jing C-B Fu C Prutsch N et al Synthetic lethal targeting of TET2-mutant hematopoietic stem and progenitor cells (HSPCs) with TOP1-targeted drugs and PARP1 inhibitors Leukemia 34 2992 3006 2020 32572188 10.1038/s41375-020-0927-5

[98] Moore KN Mirza MR Matulonis UA The poly (ADP ribose) polymerase inhibitor niraparib: Management of toxicities Gynecol Oncol 149 214 220 2018 29397193 10.1016/j.ygyno.2018.01.011

